# Curative effect observation among patients with alcohol dependence in rehabilitation period by grouping motivational interviewing

**DOI:** 10.12669/pjms.38.7.4741

**Published:** 2022

**Authors:** Yong Xu, Xiuling Pan, Chunqing Cui, Yanfeng Li, Wei Zhang

**Affiliations:** 1 Yong Xu, Department of Psychiatry, Hebei Province Veterans Hospital, Baoding, 071000, Hebei, China; 2 Xiuling Pan, Department of Psychiatry, Hebei Province Veterans Hospital, Baoding, 071000, Hebei, China; 3 Chunqing Cui, Department of Psychiatry, Hebei Province Veterans Hospital, Baoding, 071000, Hebei, China; 4 Yanfeng Li, Department of Psychiatry, Hebei Province Veterans Hospital, Baoding, 071000, Hebei, China; 5 Wei Zhang, Department of Psychiatry, Hebei Province Veterans Hospital, Baoding, 071000, Hebei, China

**Keywords:** Alcohol dependence syndrome, Motivational interviewing (MI), Psychological dependence

## Abstract

**Objective:**

To explore the effect of grouping motivational interviewing on psychological craving of patients with alcohol dependence in the rehabilitation.

**Methods:**

In this prospective study one hundred patients with convalescent alcohol dependence admitted to Hebei Province Veterans Hospital from October 2017 to June 2019 were randomly divided into two groups, the experimental group and the control group, 50 cases in each. The experimental group was administrated oxazepam as a replacement therapy and the motivational interviewing. The control group was administrated oxazepam as a replacement therapy and routine health education. Both groups continued treatment for three months. Curative effect was assessed before treatment, and two weeks, four weeks and three months after treatment by using Penn Alcohol Craving Scale (PACS), Hamilton Depression Rating Scale (HAMD) and Hamilton Anxiety Rating Scale (HAMA).

**Results:**

PACS, HAMD and HAMA in the experimental group were significantly lower than those in the control group (P < 0.05).

**Conclusion:**

Grouping motivational interviewing can effectively reduce the degree of psychological dependence on alcohol and improve the symptoms of anxiety and depression in patients with alcohol dependence during rehabilitation period.

## INTRODUCTION

Alcohol dependence is a common psychiatric disorder.^1^ In China, the prevalence of alcohol dependence has shown a gradual increase with the economic growth and the improvement of people’s living standard.^2^ The prevalence of alcohol dependence in China today is about 2.2% and 3.7% respectively in current and lifetime.^3^ Alcohol consumption is reported to be the third leading risk factor influencing the global burden of disease.^4^ The medical profession has paid great attention to the treatment of alcohol dependence syndrome, however, high re-drinking has always been a major problem in the treatment. Patients’ psychological craving for alcohol and negative emotions such as depression and anxiety are important influencing factors for the re-drinking in their rehabilitation period.^5^

In clinical studies, positive psychological intervention for patients with alcohol dependence syndrome in the rehabilitation period has been shown to effectively alleviate patients’ psychological craving for alcohol, improve the influence of negative emotions such as depression and anxiety, and thus reduce the rate of relapse.^6^ Motivational interviewing (MI) is a technique for psychological therapy, which promotes behavioral change by reinforcing the intrinsic motivation of patients,^7^ and it has been widely used in the treatment of substance abuse abroad.^8^ In China, MI has also been reported to intervene in heroin addicts, but studies on its role in alcohol dependence syndrome have been rarely reported.^9^

In this study, grouping MI was used to conduct psychotherapy on patients with alcohol dependence syndrome during the rehabilitation period, so as to observe the efficacy in improving patients’ psychological craving and negative emotions such as depression and anxiety.

## METHODS

All subjects with alcohol dependence syndrome admitted to our hospital from October 2017 to June 2019 were incuded in the study.

### Ethical approval

The prospective study was approved by the Institutional Ethics Committee of Hebei Province Veterans Hospital on December 10, 2017 (No.[2017]113), and written informed consent was obtained from all participants

### Inclusion criteria

Those meeting the diagnostic criteria for alcohol dependence syndrome described in The International Statistical Classification of Diseases and Related Health Problems 10th Revision (ICD-10); all were in rehabilitation period after drug replacement therapy and had no physical withdrawal symptoms; the patient or his/her legal guardian signing an ICF after fully understanding the content of this study; all included were male, aged 18-60 years; had primary school education or above.

### Exclusion criteria:

- Patients with serious physical diseases
- Patients with other mental diseases;
- Patients with intellectual disability or severely impaired cognitive function could not complete the interview.

A total of one hundred cases were included and randomly divided into the experimental group and the control group, 50 cases each. Patients in the experimental group had an average age of 42.24±7.77 years, an average history of drinking of 19.38±6.79 years, a PACS of 26.66±1.88, a HAMD of 34.20±8.24, and a HAMA of 29.50±8.50. Patients in the control group had an average age of 44.92±9.53 years, an average history of drinking of 21.76±9.17 years, a PACS of 26.66±1.77, a HAMD of 33.02±6.14, and a HAMA of 29.54±6.22.

In the experimental group, the patients were treated with MI in the manner of five persons/group, once a week, and the treatment was divided into four stages: 1). Introducing of mainly establishing a good therapeutic relationship; 2). Focusing of helping patients clearly identify the direction of change; 3). Arousing of the patient’s own motivation for change; 4). Planning of help patients make specific action plans. Patients in the control group were given routine health education. Both groups continued treatment for three months. Before treatment, and two weeks, four weeks and three months after treatment, patients’ craving for alcohol was assessed by using PACS; and improvement of patients’ depression and anxiety symptoms was assessed by using HAMD-24 and HAMA, respectively.^10^,^11^

### Statistical analysis

The collated data was entered into SPSS19.0, and the t test, x^2^ test and repeated measures data analysis of variance were performed.

## RESULTS

PACS of both groups before treatment, and two weeks, four weeks and three months after treatment are shown in Table-I. Repeated measures analysis of variance showed that the primary time effect was statistically significant (F=3187.669, P<0.001). The main effect between the groups was statistically significant (F=10.558, P=0.002), and the score in the experimental group was lower than that of the control group. The interaction of group × time was statistically significant (F=8.496, P<0.001). Pairwise comparison showed that the differences between each evaluation time point were statistically significant (all P<0.001), which was manifested as the PACS tendency gradually decreasing, and the decline trend of PACS in the experimental group was significantly greater than that in the control group.

**Table-I:** Comparison of PACS between the 2 groups before and after treatment.

Time	Experimental group	Control group	Time effect	Group×time	Between-group effect
Before treatment	26.66±1.88	26.56±1.77	F=3187.669 P<0.001	F=8.496 P<0.001	F=10.558 P=0.002
2-weeks after treatment	19.68±1.62	20.56±1.75			
4-weeks after treatment	13.20±2.07	14.86±2.00			
3-months after treatment	7.06±2.69	8.70±1.99			

HAMD-24 of both groups before treatment, and two weeks, four weeks and three months after treatment are shown in Table-II. Repeated measures analysis of variance showed that the primary time effect was statistically significant (F=906.114, P<0.001). The main effect between the groups was statistically significant (F=4.148, P=0.044), and the score in the experimental group was lower than that of the control group. The interaction of group × time was statistically significant (F=10.715, P<0.001). Pairwise comparison showed that the differences between each evaluation time point were statistically significant (all P<0.001), which was manifested as the HAMD-24 tendency gradually decreasing, and the decline trend of HAMD in the experimental group was significantly greater than that in the control group, indicating that the experimental group has a better curative effect than that in the control group.

**Table-II:** Comparison of HAMD between the 2 groups before and after treatment.

Time	Experimental group	Control group	Time effect	Group× time	Between-group effect
Before treatment	34.20±8.24	33.02±6.14	F=906.114 P<0.001	F=10.715 P<0.001	F=4.148 P=0.044
2-weeks after treatment	24.56±5.75	26.14±4.78			
4-weeks after treatment	15.62±5.37	17.54±3.79			
3-months after treatment	6.28±2.01	10.86±3.51			

HAMA of both groups before treatment, and two weeks, four weeks and three months after treatment are shown in Table-III. Repeated measures analysis of variance showed that the primary time effect was statistically significant (F=870.892, P<0.001). The main effect between the groups was statistically significant (F=4.417, P=0.038), and the score in the experimental group was lower than that of the control group. The interaction of group × time was statistically significant (F=7.153, P<0.002). Pairwise comparison showed that the differences between each evaluation time point were statistically significant (all P<0.001), which was manifested as the HAMA tendency gradually decreasing, and the decline trend of HAMA in the experimental group was significantly greater than that in the control group.

**Table-III:** Comparison of HAMA between the 2 groups before and after treatment

Time	Experimental group	Control group	Time effect	Group× time	Between-group effect
Before treatment	29.50±8.50	29.54±6.22	F=870.892 P<0.001	F=7.153 P=0.002	F=4.417 P=0.038
2-weeks after treatment	21.48±6.87	22.58±5.54			
4-weeks after treatment	13.30±4.82	16.44±4.73			
3-months after treatment	5.08±2.12	8.88±3.18			

## DISCUSSION

Alcohol is a psychoactive substance.^12^ In case of long-term and heavy alcohol consumption, people may develop alcohol dependence syndrome, a chronic and highly recurrent psychiatric disease.^13^,^14^ Although drug withdrawal therapy is effective in alleviating physical withdrawal symptoms, many people who recover from withdrawal will re-drink again.^15^,^16^ Data show that the patients suffering from alcohol dependence have a 56.2% relapse rate three months after discharge from the hospital. Studies have reported that psychological craving is the main factor for relapse in patients suffering from alcohol dependence.^17^,^18^

There have been reported that patients suffering from alcohol dependence still have latent psychological craving after withdrawal, and their own psychological craving and negative emotions are the main reasons for re-drinking.^19^,^20^ Therefore, psychotherapy is particularly important in the treatment of alcohol dependence. In this theme, patients with alcohol dependence syndrome during the rehabilitation period are grouped and treated by using the MI technique, and compared with the results from the control group. The results showed that the PACS, HAMD, and HAMA of the experimental group are significantly lower than those of the control group (P<0.05) at the end of two weeks, four weeks and three months after treatment. The results were similar to previous studies.^21^,^22^ This indicated that grouping MI can effectively reduce the psychological craving for alcohol of patients with alcohol dependence syndrome during the rehabilitation period, improve the patients’ negative emotions such as depression and anxiety and enhance the patient’s intrinsic motivation to stop drinking, thus has a positive effect on successful abstinence. This study further enriched the clinical research data of group motivational interview assisted treatment of alcohol dependence syndrome.

### Limitations of this study

The number of subjects included in this study is limited, so the conclusions drawn may not be very convincing. In addition, we only analyzed and discussed the cases included in our hospital, which may not be representative enough. We look forward to a multi-center study in the future to reach more comprehensive conclusions.

## CONCLUSION

Group motivational interviewing can effectively reduce the degree of psychological dependence on alcohol and improve the symptoms of anxiety and depression in patients with alcohol dependence during rehabilitation period.

### Authors’ Contributions:

**YX and** **XP** designed this study, prepared this manuscript, are responsible and accountable for the accuracy and integrity of the work. **CC** collected and analyzed clinical data. **YL and** **WZ:** Data analysis, significantly revised this manuscript.
